# Effective Vehicle-Based Kangaroo Detection for Collision Warning Systems Using Region-Based Convolutional Networks

**DOI:** 10.3390/s18061913

**Published:** 2018-06-12

**Authors:** Khaled Saleh, Mohammed Hossny, Saeid Nahavandi

**Affiliations:** Institute for Intelligent Systems Research and Innovation (IISRI), Deakin University, Waurn Ponds, Victoria 3216, Australia; mohammed.hossny@deakin.edu.au (M.H.); saeid.nahavandi@deakin.edu.au (S.N.)

**Keywords:** kangaroo collision, kangaroo detection, collision avoidance, kangaroo dataset

## Abstract

Traffic collisions between kangaroos and motorists are on the rise on Australian roads. According to a recent report, it was estimated that there were more than 20,000 kangaroo vehicle collisions that occurred only during the year 2015 in Australia. In this work, we are proposing a vehicle-based framework for kangaroo detection in urban and highway traffic environment that could be used for collision warning systems. Our proposed framework is based on region-based convolutional neural networks (RCNN). Given the scarcity of labeled data of kangaroos in traffic environments, we utilized our state-of-the-art data generation pipeline to generate 17,000 synthetic depth images of traffic scenes with kangaroo instances annotated in them. We trained our proposed RCNN-based framework on a subset of the generated synthetic depth images dataset. The proposed framework achieved a higher average precision (AP) score of 92% over all the testing synthetic depth image datasets. We compared our proposed framework against other baseline approaches and we outperformed it with more than 37% in AP score over all the testing datasets. Additionally, we evaluated the generalization performance of the proposed framework on real live data and we achieved a resilient detection accuracy without any further fine-tuning of our proposed RCNN-based framework.

## 1. Introduction

Recently, the kangaroo detection problem on Australian traffic environments has received some attention from both research communities and car manufacturers—specifically, with the recent reports of the increased number of accidents happening on the Australian roads due to collision between Kangaroos and human-driven vehicles [1]. The conflict not only exists between human-driven vehicles and kangaroos. According to recent media releases, highly and fully automated vehicles tested by major car manufacturers on Australian roads are also challenged by the same problem [2]. Ultimately, the efforts that have been made to prevent or reduce the number of collisions happening between vehicles and other wild animals such as deer and moose could be categorized into two main categories, namely road-based techniques and vehicle-based techniques. Road-based techniques are the traditional prevention measures such as fences and wildlife reflectors that have been deemed to be insufficient for reducing the number of collisions as it was shown in [3].

On the contrary, vehicle-based techniques rely either on passive sensors externally mounted on top of the vehicles or on the tremendous amount of active sensors that exist nowadays in most of the newly released vehicles in order to deter or detect wild animals. External passive sensors mounted on the vehicles that were used to keep wild animals away from the vehicles include high pitched signal whistles and tungsten halogen (TH) lamps [4,5]. Active sensors used for detecting wild animals are such as optical sensors (cameras: monocular, stereo, thermal and Light Detection and Ranging (LIDAR)) or radar [6,7]. Due to the limitations of vehicle-based techniques relying on passive sensors such as the necessity for a visible line of sight between the wild animals and the vehicle, and their higher rate of false alarms, active sensors-based techniques were shown to be more effective as it was discussed in [8]. Another major advantage of using active sensors in vehicle-based techniques for wild animals’ collision detections, specifically the optical ones, is their wide range of covering the road and its sides in addition to their relatively higher accuracy when compared to passive sensors as well as other active sensors (i.e., radars). Thus, in this work, we will be focusing more on the vehicle-based techniques relying on active sensors.

In the literature, for almost all of the vehicle-based techniques relying on active sensors for the detection of wild animals such as deer and moose, their methodology can be broken down into two main consecutive stages. The first stage is feature extraction, whereas a hand-crafted set of visual/appearance features are extracted from colour and/or thermal images. The second stage is the classification stage, whereas the combined extracted features from the previous stage became the basis for training an object classifier that can detect the class of wild animals of interest at testing time [6,7,9].

One of the drawbacks of the aforementioned approach is that it needs a lot of iterations and fine-tuning in order to determine what are the most effective features to be considered in the first place. Moreover, the separation that happens between the two stages (i.e., features extraction and classification) make it inefficient to accommodate any new features or training data in the future.

On the other hand, data-driven approaches such as convolutional neural networks (ConvNets), do not suffer from these problems that conventional methods are challenged with because they can be trained in an end-to-end fashion which alleviates the need for handcrafting specific features. In addition, ConvNets have achieved state-of-the-art results in different tasks, especially when it comes to vision-based tasks such as object classification, object detection and semantic segmentation [10,11,12]. However, they still require a handful amount of labeled data for training and validating their operation, which in our case is hard to collect for wild animals in general and for our wild animal of interest (i.e., kangaroo) specifically. Additionally, the annotation process is a cumbersome and time-consuming task.

One of the approaches that have been explored recently to tackle the scarce amount of labeled data for training ConvNets is the utilization of photo-realistic simulation framework in order to generate labeled data for training and validating ConvNet models [13,14]. In our formulation for the kangaroo detection problem, we will adopt a similar methodology to generate an annotated synthetic depth images of highway traffic scenes containing kangaroo instances within them using the data generation pipeline proposed in [15]. Then, using the generated labeled data, we will utilize one special variant of ConvNet that have achieved state-of-the-art results in real-time object detection benchmarks, faster region-based convolutional neural networks (Faster RCNN). Our contribution in this work can be summarized into the following:

- A novel large annotated synthetic depth images dataset of kangaroos in highway traffic environment roads similar to Australian roads. To our knowledge, no other dataset for kangaroos similar to it exists in the literature.
- A ConvNet model based on Faster RCNN architecture trained on synthetic depth images that can accurately detect and localize any instances of kangaroos in synthetic depth images.
- A ConvNet model for kangaroo detection trained only on synthetic depth images that can generalize robustly to detect and localize kangaroo instances in real depth images.

The rest of the paper will be organized as follows: Section 2 gives an overview of the work in the literature related to the vehicle-based techniques relying on active sensors for wild animal detection. In Section 3, the formulation of the kangaroo detection problem will be introduced and our proposed methodology will be discussed. Section 4 describes the data generation pipeline used for generating both the training and testing dataset for the kangaroos. In Section 5, our proposed framework for the kangaroo detection task will be discussed, and the details of both its architecture and training procedure will be presented. In Section 6, the results of our experiments using our proposed kangaroo detection framework on the generated synthetic testing datasets and the real collected depth images will be evaluated and discussed. Finally, conclusions and future work are presented in Section 7.

## 2. Related Work

In the literature, the wild animal detection problem in general and the kangaroo detection specifically in the context of vehicle-based collision avoidance systems have not been thoroughly explored as opposed to other vulnerable road users such as pedestrians or cyclists [16,17]. In [6], one of the early works on vehicle-based wild animal detection was proposed. Their wild animal of interest was moose, whereas they introduced infrared-based vision system for detecting moose as an early warning collision system. They employed an Icelandic horse with a rider on top of it as a thermal replica for the moose and used a simple threshold-based detection with adaptive mean-shift tracking algorithm in order to detect and track the thermal replica acting as moose. They tested their system on nine simple scenarios in a controlled environment, and, for most of them, the thermal replica was stationary on a predefined distance. One of the major drawbacks of this system was its reliance on a hard-coded thermal threshold value corresponding to the thermal information of moose, which is so dependent on the type of the thermal camera being used and extremely hard to find a certain one that can be generalized across different environments.

Zhou et al. [18] presented another infrared-based system for deer detection by relying on the Histogram of Oriented Gradient (HOG) algorithm [19] as a feature descriptor/extractor and the Support Vector Machines (SVM) as a classifier. Their technique operates in a sliding window fashion, whereas they firstly apply a contour-based stage in order to filter out the most probable regions that are not corresponding to deer in the input image, and then a sliding window will be go over the filtered regions and calculate HOG features locally. Finally, the SVM classifier is used to determine whether each window region contains a deer or not. Once again, similar to [6], one of the main drawbacks of this proposed methodology is that it relies on a threshold value for a contour-based stage that requires a lot of effort to determine. Additionally, since they rely on a sliding window to go over a non-perfect filtered region, it suffers from a higher false positive rate.

Most recently, Mammeri et al. [9] presented a vision-based system for detecting large animals such as moose and deer. They relied on their approach on a combination of feature extractors such as HOG, local binary pattern (LBP) and Haar in conjunction with two consecutive classifications stages based on AdaBoost and cascaded AdaBoost. The extracted features from the three aforementioned extractors are used as a weak classifier in the first AdaBoost stage to discriminate between the features that have a positive large animal in them and the negative ones. The weak classifiers corresponding to the features from HOG and Haar determine whether the features are positive or not based on a predefined threshold value. The weak classifier for the LBP feature extractor, on the other hand makes its decision based on the 245 branches of its internal decision tree. During the cascaded AdaBoost classification stage, the output from all the individual weak classifiers is linked together in order to get better detection accuracies and reduce computation time.

Despite the improvements that have been achieved when compared to [18] by using the combination of feature extractors with the cascaded AdaBoost classifier, this approach still requires many more threshold values to be predefined in advance. It is also worth noting that, in this approach, the data that have been collected for training, testing and tuning the classifiers’ threshold values were only considering lateral views of the large animals when fully observable at daytime on the driving path. Since this is not likely the case with kangaroos, as collisions between them and vehicles usually happen at dusk and night times with kangaroos coming from either sides of the road, this approach would not be the optimal approach for the kangaroo detection problem.

## 3. Proposed Methodology

In the previous section, we have discussed most of the vehicle-based approaches presented in the literature for the wild animal detection in the traffic environments problem. Despite how powerful these approaches are, they may not be the suitable approach when it comes to solving the kangaroo detection problem.

### 3.1. Problem Formulation

Unlike other wild animals such as deer and moose, kangaroos inhibit much more rapid and unpredictable movements in road and traffic scenes. Thus, any proposed vehicle-based approach to tackle this problem needs to take into account the different activities done by the kangaroo in traffic road scenes and their different viewpoints when observed by any vehicle-based optical sensor. Given that the conventional approaches proposed in the literature rely on hand crafting specific features from each activity/scenario that are discriminative enough for a classification stage. Thus, these approaches will necessitate designing numerous individual classification stages for each activity/scenario for the kangaroo detection problem that would be inefficient in terms of scalability and real-time performance, which is a critical requirement for any vehicle collision avoidance system.

In our formulation for the kangaroo detection problem, similar to the prior work on wild animal detection, we will be also pursuing a vehicle-based technique relying on active sensors (stereo cameras or 3D LIDAR). However, rather than explicitly extracting a specific feature for each possible activity/scenario performed by the kangaroo in traffic and road environments, we will utilize a data-driven approach based on Convolutional Neural Networks (ConvNets). More specifically, for the task of kangaroo detection, we will be utilizing the state-of-the-art data generation pipeline introduced in [15] to generate synthetic depth images of highway traffic scenes containing kangaroo instances annotated with bounding boxes inside them. The rationale for using depth images rather than monocular RGB/thermal images is twofold, depth information is critical for any collision avoidance or assessment systems, and, in order to obtain it from monocular RGB cameras, is not as reliable as from stereo cameras or 3D LIDARs [20,21]. On the other hand, thermal cameras cannot provide any depth information and they have major heat issues that make them not a reliable solution for collision avoidance systems. The second reason is the convenience to achieve depth images from different sensors shipped nowadays with intelligent vehicles whether they are low-cost ones such as long-range stereo cameras or the expensive ones such as 3D LIDARs.

Given the annotated generated depth image dataset, we can build and train a ConvNet model based on one of the successful deep ConvNet architectures for object localization and detection, Region-based Convolutional Neural Networks (RCNN) [22]. By training an RCNN-based model in an end-to-end fashion, we overcome the necessity for manually designing many specialized feature descriptors and extractors that were needed in other traditional techniques. However, we will be utilizing the expressiveness of the hierarchal representation learning of ConvNets that will address the aforementioned challenges [23].

### 3.2. Region-Based Convolutional Neural Networks

Recently, ConvNets have been achieving state-of-the-art results on a number of computer vision tasks such as object classification, object detection and semantic segmentation [10,11,12]. One of the best excelling architectures for object localization detection tasks is Region-based convolutional neural networks (RCNN) [22]. RCNNs are comprised mainly of two parallel modules which together form a multi-task learning model for object detection that can be trained jointly in an end-to-end fashion.

The backbone module of the RCNN architecture is the typical ConvNet model used in image classification tasks, which consists of three main stacked and interleaving layers, namely convolution layers, pooling layers and fully connected layers. Convolution layers act as an automatic feature extractor and each layer of them produces unique feature maps attained spatially from various locations in their input data. Following each convolution layer, a rectified linear unit (ReLU) is typically existent in order to inject nonlinearity between the elements of the convoluted feature maps [23]. Pooling layers, on the other hand, act on the output feature maps from convolution layers and down-sample them spatially in order to minimize the total number of weights to be learned. Most commonly used pooling operations done in pooling layers are max pooling operations. In fully connected (FC) layers, neurons of their input feature maps are linked together with their internal neurons. The difference between fully connected and convolution layers is that their internal neurons are not spatially collated.

The other module of RCNNs is called a region proposal module, which yields a set of unique regions called RoIs in the form of rectangle windows from the input image. The RoIs are utilized at an intermediate stage by the backbone module of RCNN, which was described previously through a dedicated pooling layer, called RoI pooling layer. The feature maps outputted from the RoI pooling layer are then vectorized into a 1D feature array by the FC layer of the backbone module of RCNN. Finally, at the last layer of RCNN, the objective training function exists, which is a joint multi-task loss layer. Additionally, a softmax and regression layer follow, which are responsible for scoring object classes and regressing the positions of an object’s bounding box, respectively.

There are two variants of RCNN architectures and the main distinction between the two lies in how the proposal of regions are generated. The first variant was the earliest one introduced in [24], which relies on a selective search (SS) technique. The selective search was utilized as a preparation stage to extract a set of RoI for the backbone module of the RCNN. The aforementioned variant has provided decent performance in general, albeit the computational complexity of SS technique on CPU influenced the performance of its real-time capabilities. In order to overcome such problem, the other variant, called Faster RCNN [22], was proposed to fix the specific issue of slower real-time performance, and hence the name Faster. In Faster RCNN (FRCNN), the region proposal network (RPN) was introduced. RPN replaced the computationally expensive selective search stage of RCNN by merging it within the backbone module of the RCNN. RPN is comprised of convolutional layers only, whereas the early layers are shared with the aforementioned RCNN’s backbone module.

## 4. Data Generation Pipeline for Kangaroo Detection

In order to train, validate and test any ConvNet-based model, a handful of annotated data is required. Given the problem at hand of kangaroo detection, getting a large amount of real data of them that can capture all their movements and activities in traffic environments is not an easy task. Moreover, the process for annotating them with bounding boxes is a time-consuming process. On the other hand, one of the recent cost-effective and efficient paradigms that have been explored to overcome the scarcity of annotated data for ConvNets is the use of synthetic data images generated from photo-realistic simulation and/or game engine frameworks [13,14].

Similarly, we will utilize the simulation-based data generation pipeline proposed in [15]. Based on this pipeline, a huge amount of synthetically generated depth images annotated with kangaroos in traffic environments similar to the ones on Australian roads can be achieved. The data generation pipeline itself is based on Blensor [25], a simulation framework for various types of range scanners. In the following subsections, we will present the main components that constitute the data generation pipeline being used for generating data for the kangaroo detection problem.

### 4.1. Motion Capture and Mapping

**Figure 1:**
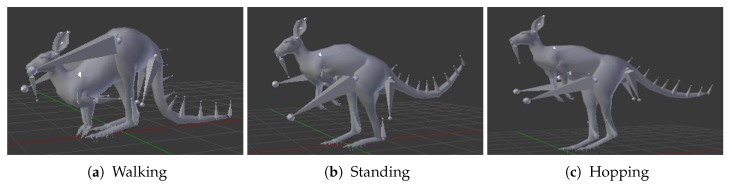
The three MoCap activities used for the generated kangaroo dataset mapped on a kangaroo model.

Firstly, a motion capture system (MoCap) is utilized to record a dataset of three distinctive activities performed by kangaroos in real life, namely standing, hopping, and walking. In Figure 1, a demonstration of these three activities is shown. Afterward, we map the MoCap data on a 3D simulated kangaroo model with its different anthropometric measures inside Blensor [25].

### 4.2. Scene Modelling and Depth Images Rendering

We designed inside Blensor [25] four different scenes of traffic highway roads with versatile objects for instance buildings, vehicles, motorcyclists, trees and electricity poles. Consequently, for each 3D model of kangaroos inside each scene, we apply a motion trajectory comprised of a combination of the three MoCap data described earlier with more focus on the hopping activity since it is the most common activity done by kangaroos in traffic environments. In each scene, we made sure that all motion trajectories done by the kangaroos mimic the most commonly experienced scenarios of collisions between kangaroos and vehicles, such as when a kangaroo is walking or standing on the side of the roads or behind trees and then suddenly does a series of hopping activities to cross the road.

For rendering the scene and generating the depth images, we made a camera setup of four dynamic virtual range cameras mounted in the scene on a height similar to the height of vehicle-based cameras (roughly 1.5 m). The four cameras were dynamically flexible so that they could follow the kangaroo instances in the scene with a constraint of being on a variable distance ranging from 10 m to 50 m away from the kangaroos. Therefore, the kangaroos were tracked in the scene from four different viewing angles, namely the back, front and two side views. As a result, during the simulation session, a depth image containing at least one kangaroo model with the other objects in the scene was obtained at each time-instance from each virtual camera. Finally, with the help of our developed software plug-in tool inside Blensor, the scene was scanned twice from each virtual camera from the four cameras. The first scan was with only the kangaroo instances rendered from the scene. In the second scan, however, the rest of the scene’s background objects were rendered. Consequently, we achieved more precise bounding box labels of all kangaroos in the scene in each camera of the 4 = four virtual ones.

### 4.3. Depth Image Dataset Pre-Processing and Preparation

By following the rendering procedure previously discussed, we obtained from the four modeled scenes a total of 17K depth images containing from one to four kangaroo instances annotated in each depth image. We split the dataset into two with 12,000 depth images for training and 5000 for testing, and we made sure that the training and testing dataset splits are spanning two different scenes from the total of four scenes used in the data generation process.

One of the known issues with depth images when feeding them to ConvNet-based models is the low contrast property of depth images [26,27]. The early layers of ConvNets are trying to find a set of features that are discriminative enough to be picked up in any generic RGB images such as the edges between foreground and background objects. Since these low features are not that prevalent in depth images, to overcome this problem, we colorized the single channel generated depth images using the procedure presented in [26].

**Figure 2:**
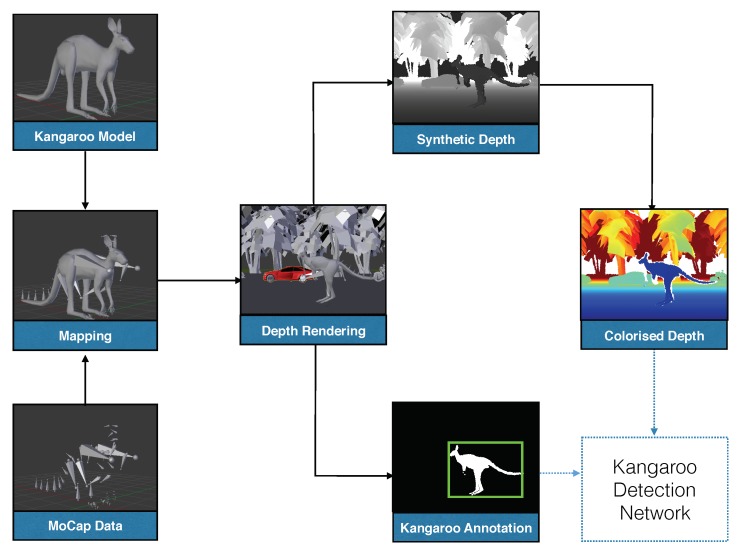
Our pipeline for data generation for the kangaroo detection task. Firstly, the mapping of MoCap data on 3D kangaroo model(s) is performed. Highway and urban traffic scenes are then modeled and merged with animated kangaroos for rendering. Colorized and bounding box annotation of kangaroo instances are then generated.

Additionally, in order to simulate some of the realistic properties existing in real depth and range images such as the ones obtained from stereo cameras or other range sensors, we introduced some noise and distortions to the perfectly generated synthetic depth images from our data generation pipeline according to the noise model introduced in [25,28]. Applying those kinds of noise and distortions can also be viewed as a type of data augmentation that was proved to both enhance the performance of the trained ConvNet-based models and reduce the effect of overfitting during training [10]. The full data generation pipeline we used for generating the 17K kangaroo depth image dataset is illustrated in Figure 2.

## 5. Faster R-CNN for Kangaroo Detection

**Figure 3:**
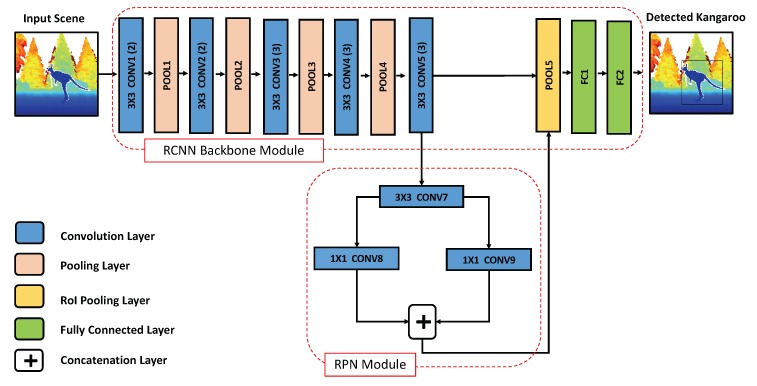
Proposed faster region-based convolutional neural network framework for the kangaroo detection task. The network’s input is a colorized depth image of size (640 W × 480 H × 3). The network output is the coordinates of the the localized bounding boxes of each kangaroo instance in the scene.

As we discussed in Section 3.2, there are two derivatives of RCNN for object detection tasks, with the only major change being how the region proposal module actually works. In light of the advantages we presented earlier regarding how FRCNN is considered more efficient architecture than the other traditional RCNN architecture especially when it comes to the real-time performance, we find that the FRCNN architecture would be more suitable for the task of kangaroo detection in traffic environments (illustrated in Figure 3). In the following subsections, we will discuss the details of our proposed FRCNN-based architecture for the kangaroo detection problem.

### 5.1. Network Architecture

Our proposed FRCNN-based model for the task of kangaroo detection will be following the same organization as the original FRCNN network in [22]. However, the dimensionality of the input images in our proposed architecture will be different, whereas ours will be (480 H × 640 W) rather than the (600 H × 1000 W) of the original FRCNN network. The input images will be depth images colorized and prepared according to the procedure discussed in Section 4, The output from the proposed network is a similar image to the input one, but with any kangaroo instances in the input images detected and localized. The kangaroo instances are localized with their bounding boxes coordinates in the input image along with the detection confidence of the network prediction. The first stage of the network, will be the backbone ConvNet module of the RCNN. In our proposed network, the backbone module will be consisting of 13 (convolution+ReLU) layers interleaved with max pooling layers similar to the VGG-16 network [29].

The 13 convolution layers of the backbone module will be shareable with the other module of our proposed RCNN-based network, the RPN module. The input to this module is the last convolution layer’s feature maps from the RoI pooling layer of our backbone module. The RPN module consists of three other convolution layers, which are responsible for a generation of a set of region proposals representing different objects in the input image, and, in our case, the total number of these proposals is 300. The extraction of the regions’ proposals is done automatically over the feature maps from the last convolution layer of the backbone module, just by sliding a set of bounding boxes over these feature maps with different *n* scales and *m* aspect ratios. The variables *n* and *m* are hyper-parameters for the network and were set similar to the original FRCNN network with 3 for both scales (0.5, 1, 2) and aspect ratios (2:1, 1:1 and 1:2). The multiplication of *n* and *m* in the FRCNN architecture is called “anchors”.

One of the advantages of using anchors in our proposed network is that they provide an inexpensive method that is translation-agnostic to the different sizes and shapes of the class of objects need to be detected (i.e., kangaroos) in the input images. Thus, it alleviates the need for training a different cascading number of individual classifiers for each possible scale and aspect ratio of the kangaroo, which was a necessity for the other proposed approaches’ literature for other wild animal detection problems [9,18]. The extracted region proposal from the RPN module is then passed along with the feature maps from the last convolution layer of the backbone module to the Region of Interest (RoI) pooling layer. The RoI pooling layer is a max pooling layer that down-samples the number of its input feature maps to a fixed size (7 × 7 in our case), which helps in accelerating the training and testing time of the proposed network and it also allows for training the RCNN network with its two internal modules jointly in an end-to-end fashion. Finally, the output down-sampled feature maps from the RoI pooling layer are fed to the last two layers of the backbone module, which are two fully connected (FC) layers.

### 5.2. Network Training

The training of any ConvNet-based model can be viewed as an optimization problem, whereas, using a learning algorithm, the objective is to minimize a given and known loss function. In our proposed FRCNN-based network for the kangaroo detection problem, we have a two loss functions that are required to be trained jointly. The first loss function is $L_{RPN}$, the loss function of the RPN module of our proposed FRCNN-based network. The second loss function is $L_{FRCNN}$, which is the loss function of the backbone module of the FRCNN network. The total loss function $L_{KANG}$ that needs to be optimized is just the sum of both $L_{RPN}$ and $L_{FRCNN}$.

The loss function for $L_{RPN}$ is calculated according to the RPN loss function proposed in [24] as follows:

(1) $$\begin{array}{cc} L_{RPN}\left(p_{a},t_{a}\right) & =\frac{1}{N_{RPN\_cls}}\sum_{a}L_{RPN\_cls}\left(p_{a},p_{a}^{*}\right)\\ & +\frac{1}{N_{RPN\_reg}}\sum_{a}p_{a}^{*}L_{RPN\_reg}\left(t_{a},t_{a}^{*}\right), \end{array}$$

whereas *a* is the index of anchors and $p_{a}$ is the RPN’s module predicted classification probability of the *a*-th anchor to be a kangaroo. $N_{RPN\_cls}$ and $N_{RPN\_reg}$ are normalization factors for stable optimization requirements and we set them equal to the mini-batch size of 128. $L_{RPN\_cls}$ is classification loss of RPN, which is the log loss of binary classification task (either a kangaroo or not). On the other hand, $p_{a}^{*}$ is the ground truth label of the *a*-th anchor, which is one if positive and zero otherwise. $L_{RPN\_reg}$ is regression loss of the RPN’s bounding box which is the smoothed $L_{1}$ loss ($t_{a}-t_{a}^{*}$) utilized in [24]. $t_{a}$ is the predicted bounding boxes’ coordinates vector ($t_{x},t_{y},t_{w},t_{h}$) for the *a*-th anchor, and $t_{a}^{*}$ is the ground truth labeled values for the positive anchor’s bounding box position only. The term ($p_{a}^{*}L_{RPN\_reg}\left(t_{a},t_{a}^{*}\right),$) is the regression loss of RPN’s bounding box, which is active for only positive *a* anchors.

The loss function for $L_{FRCNN}$ is calculated according to the backbone module loss function of FRCNN proposed in [22] as follows:

(2) $$L_{FRCNN}\left(p_{FRCNN},t_{FRCNN}\right)=L_{cls}\left(p_{rFRCNNcnn},p_{FRCNN}^{*}\right)+p_{FRCNN}^{*}L_{reg}\left(t_{FRCNN},t_{FRCNN}^{*}\right),$$

whereas $p_{FRCNN}$ is the probability of the object being kangaroo , $p_{FRCNN}^{*}$ is the label of kangaroo class, which is one in case it is positive and zero otherwise. $t_{FRCNN}$ is the predicted bounding boxes’ coordinates vector ($t_{x},t_{y},t_{w},t_{h}$) for the kangaroo object class, and $t_{FRCNN}^{*}$ is the actual label of the kangaroo’s bounding box position values. $L_{cls}$ is the loss of the classification task. $L_{reg}$ is the regression loss of the bounding boxes.

In order to jointly minimize the previous loss functions, we used the stochastic gradient descent (SGD) for training our proposed FRCNN-based network with the following hyper-parameters: mini-batches of 128; with learning rate of 0.0001 for 30K iterations; and with momentum of 0.9 and weight decay of 0.0005.

## 6. Experimental Results

In order to evaluate the performance of our proposed framework for the task of kangaroo detection quantitatively, we generated a total of 5000 synthetic depth images according to the procedure discussed in Section 4. Furthermore, in order to evaluate the performance of our proposed framework in real-life scenes of kangaroos and examine the feasibility our proposed framework to generalize to other real and live unseen scenes of kangaroo, we collected more than 250 real depth images of kangaroo in an urban traffic environment using the Microsoft Kinect sensor during night time. In the following sections, we will discuss the results of our proposed framework quantitatively and qualitatively on the generated synthetic depth testing image dataset, and qualitatively on the real live kangaroo collected depth images.

### 6.1. Comparative Results on the Synthetic Testing Dataset

We trained the other two baseline approaches to compare our proposed framework against them. The first baseline is one of the most proposed approaches in the literature for the task of wild animal detection in general, and it is a combination between feature extractor, HOG and the SVM classifier, which is pretty similar to the ones proposed in [9,18] for deer and moose detection, respectively. We refer to this technique as (HoG-SVM).

The second baseline is a similar approach to our FRCNN-based framework, however with a shallower backbone module architecture based on the VGG-CNN-M architecture proposed in [30]. The VGG-CNN-M, unlike the 13 convolution layers of VGG16 of our backbone module, consists only of five convolution layers. We refer to this technique as (FRCNN-VGG-M).

In Table 1, we report the results of the aforementioned two methods (HoG-SVM and FRCNN-VGG-M) for the kangaroo detection task against our proposed framework (FRCNN-VGG-16). We evaluated the results according to the well-known average precision (AP) evaluation metric, which was used in a number of object detection benchmarks such as the PASCAL object detection challenges [31]. The AP measure is used to describe the performance of precision/recall curve.

Due to the computational limitations of HoG-SVM based approach to obtain a trained model using the 15,000 synthetic depth images of our generated training dataset. We trained three individual HoG-SVM models on three subsets of the training dataset. The three subsets were covering the orientation of the kangaroos from the perspective of the simulated scanning range sensor. We defined the three subsets according to the following:

- Side view subset: All images that contain any kangaroo instances observed from their side view either with an orientation towards right or left from the range sensor’s perspective.
- Frontal view subset: All images that contain only front views of kangaroo instances from the perspective of the range sensor.
- Back view subset: All images that contain only back views of kangaroo instances from the perspective of the range sensor.

The reported AP scores for each trained HoG-SVM model is evaluated with the corresponding image subset in the testing image dataset with the column names (“Side”, “Frontal”, “Back”) of Table 1. Since the two FRCNN-based trained models were trained on GPU, they do not suffer from this computational constraint. Thus, our proposed frameworks FRCNN-VGG-16 and FRCNN-VGG-M were trained on the total 15,000 amount of the training dataset. However, in order to have a meaningful comparison between them and the HoG-SVM based trained models, we tested them on each subset divided according to the aforementioned method. Additionally, we have tested the three HoG-SVM trained models on the total testing dataset as stages, and reported three parallel classification stages and reported their AP measure in comparison to the other two FRCNN-based models under the column name “All” of Table 1.

**Figure 4:**
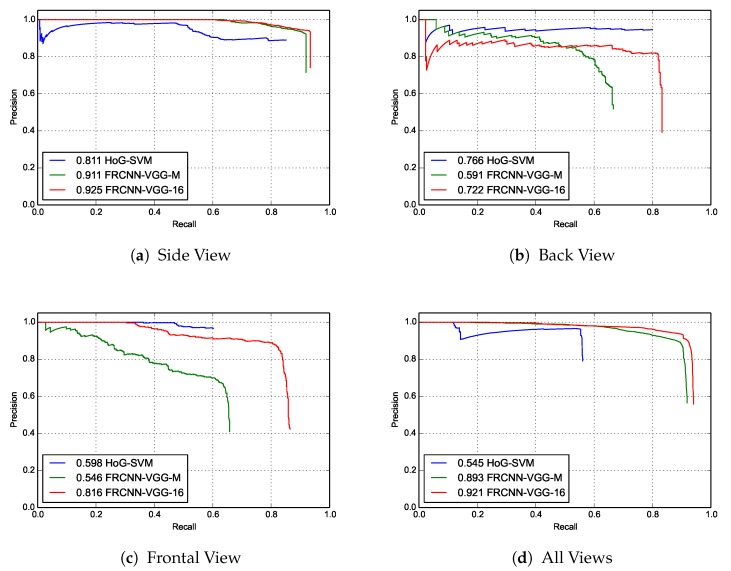
Precision–recall curves of three different object detection techniques for the kangaroo detection task over four subsets of the generated synthetic testing dataset. The score of average precision (AP) is listed before the name of each technique. The subsets are categorized according to three individual orientations of the kangaroos in the testing scenes from the range sensor’s point of view. (**a**) side view, (**b**) back view, (**c**) frontal view. Additionally, the combination of all these scenarios (**d**) all views.

As it is shown from the AP scores and the precision-recall curves in Table 1 and Figure 4 respectively, our proposed framework (FRCNN-VGG-16) for the kangaroo detection achieved resilient results in terms of AP scores and precision-recall curves over all the testing dataset. Precision–recall curves are used to indicate the tradeoff between precision and recall for different threshold values. A high area under the curve represents both high recall and high precision, where high precision relates to a low false positive rate, and high recall relates to a low false negative rate. Our approach has achieved AP score of 92.1% with more than 37% and 2% improvement over the Hog-SVM models and the FRCNN-VGG-M model, respectively. Nevertheless, our FRCNN-VGG-16 model achieved better results than the individual HoG-SVM models for both the side and frontal view kangaroo scene subsets with an improvement of 21.8% and 11.4%, respectively. On the other hand, the Back view trained HoG-SVM model achieved a better AP score on the back view subset of the testing images with only 4.4%. The justification for the marginally better performance of the HoG-SVM model over our proposed framework is since the total number of images containing back views of kangaroo instances in the total testing dataset is much lower than the other views, its lower AP score in the Back view subset.

**Figure 5:**
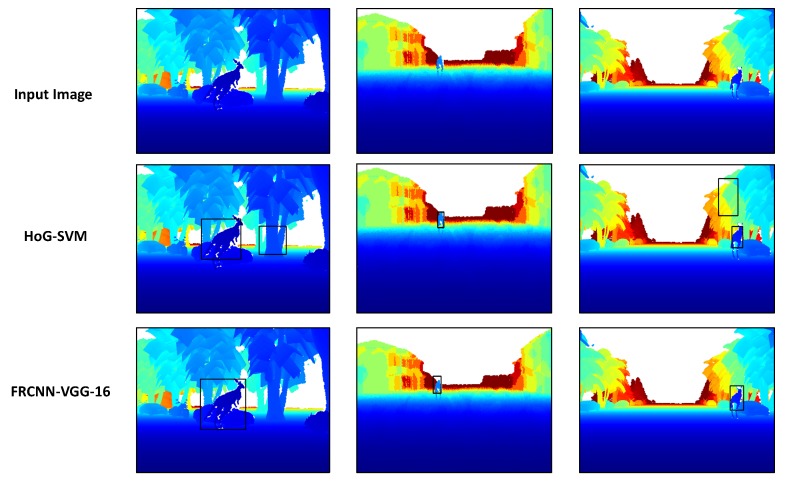
Evaluation on the synthetic test split data. **Top**: the input colorized synthetically generated depth images. **Middle**: output of the baseline HoG-SVM models. **Bottom**: prediction of proposed FRCNN-VGG-16 kangaroo detection network.

In Figure 5, some qualitative results of our proposed framework (FRCNN-VGG-16) in comparison to the HoG-SVM model over a sample from each subset from the three subsets. As it can be seen, our proposed FRCNN-VGG-16 model has superior results over the HoG-SVM model that has more false positive detections in both the side view (first image from left in the middle row) and the frontal view (third image from left in the middle row).

In Table 1, we are further reporting the real-time performance of our proposed models, which is essential for any vehicle collision avoidance systems in comparison to the HoG-SVM model in terms of frame per second (FPS) rate. As it can be seen, we have a modest real-time performance with a 9 and 17 FPS rate, respectively, whereas the HoG-SVM model achieved only 4 FPS. It is worth noting here that our proposed models were utilizing the Nvidia GeForce GTX Titan X GPU for their operation, while the HoG-SVM model was only utilizing the CPU (Intel Core i7 @ 3.40 GHz, 16 GB RAM).

### 6.2. Comparative Results on Collected Real Depth Images

For examining how our proposed framework would behave when tested on real live data of kangaroo scenes, we collected more than 250 real depth images containing kangaroo in an urban traffic environment using a Microsoft Kinect during night time. Furthermore, we have manually annotated all the kangaroo instances in all the 250 depth images with bounding boxes for a quantitative AP measure. The collected data have covered similar viewpoints of the kangaroos as the ones used in the synthetic data generation phase. It is worth noting that the collected real data were tested using our proposed models that were trained using the generated synthetic data only. In Table 1, we are reporting the AP score measure of our proposed models along with the HoG-SVM model over the real depth testing images.

**Figure 6:**
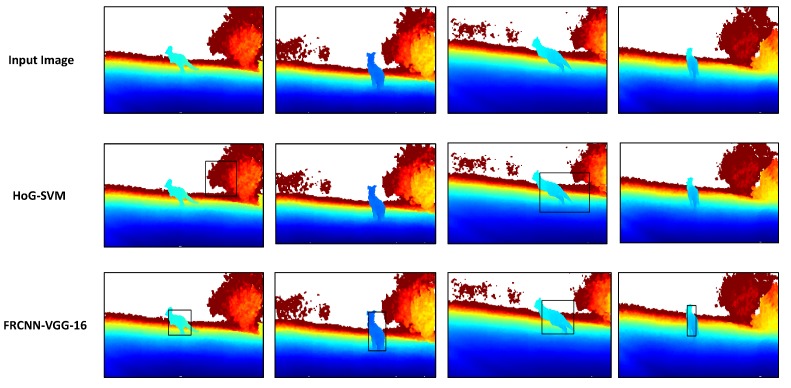
Qualitative results on real live kangaroo dataset. **Top**: the input colorized real depth images. **Middle**: Output of the HoG-SVM models. **Bottom**: output of proposed FRCNN-VGG-16 kangaroo detection network

In Figure 6, some qualitative results over the real live kangaroo dataset are shown. We also compared the performance of our proposed framework (second row from top) against HoG-SVM models (third row from top), and, as we can see, our proposed framework model can generalize pretty well, given it has not been trained on any of real live depth image datasets. Moreover, our proposed framework has achieved much better detection accuracies over the HoG-SVM models, which were either incapable of identifying the kangaroo instance in the first place (such as in second and fourth images in the middle row) or they provide inaccurate detections (such as in first and third images in the middle row).

## 7. Conclusions

In this paper, we have proposed a framework for the problem of kangaroo detection in urban and highway traffic environments based on fast object detection architecture and region-based convolution networks (RCNN). In order to overcome the scarcity of labeled data for kangaroos in traffic environments, we generated 17,000 synthetic depth images of traffic environments with kangaroo instances and annotated them was trained using our data generation pipeline. Our proposed Faster RCNN-based framework for the kangaroo detection problem on a subset of the generated synthetic depth images, and tested it on other different subsets, whereas we achieved a superior result over other two baseline models. We scored more than 92% in the AP score over the testing dataset with more than 37% improvement over one of the most utilized approaches for the wild animal detection in the literature. Furthermore, we collected more than 250 real depth images and our proposed framework have achieved a significant generalization capability with better detection accuracies when compared to the other baseline model. In our future work, we will focus on testing our proposed framework on more real live depth images of kangaroo instances in more diverse traffic environments and explore the possibilities of implementing it on a low-cost embedded GPU that could be mounted on vehicles.

## Figures and Tables

**Table 1:** Performance of the different methods on both the synthetic and real depth testing datasets according to the average precision (AP) (%) evaluation metric and the number of frame per second (FPS). The synthetic depth testing datasets were divided into four subsets for evaluation based on the kangaroo orientation from the perspective of the range sensor.

Method	Synthetic Depth (AP%)				Real Depth (AP%)	FPS (#)
	**All**	**Side**	**Frontal**	**Back**	**All**	**All**
HoG-SVM [19]	54.5	81.1	59.8	**76.6**	20	4
FRCNN-VGG-M	89.3	91.1	54.6	59.1	54.7	**17**
FRCNN-VGG-16	**92.1**	**92.5**	**81.6**	72.2	**86.2**	9
